# New Therapeutics for Heart Failure Worsening: Focus on Vericiguat

**DOI:** 10.3390/jcm13144209

**Published:** 2024-07-19

**Authors:** Patrizia Russo, Laura Vitiello, Francesca Milani, Maurizio Volterrani, Giuseppe M. C. Rosano, Carlo Tomino, Stefano Bonassi

**Affiliations:** 1 Department of Human Sciences and Promotion of the Quality of Life, San Raffaele University; Via di Val Cannuta 247, 00166 Rome, Italy; laura.vitiello@uniroma5.it (L.V.); francesca.milani@uniroma5.it (F.M.); maurizio.volterrani@sanraffaele.it (M.V.); giuseppe.rosano@uniroma5.it (G.M.C.R.); stefano.bonassi@uniroma5.it (S.B.); 2 Clinical and Molecular Epidemiology, IRCCS San Raffaele Roma, Via di Val Cannuta 247, 00166 Rome, Italy; 3 Cardiology Rehabilitation Unit, IRCCS San Raffaele Roma, Via della Pisana 235, 00163 Rome, Italy; 4 Cardiology, San Raffaele Cassino Hospital, Via Gaetano di Biasio, 1, 03043 Cassino, Italy; 5 Scientific Direction, IRCCS San Raffaele Roma, Via di Val Cannuta 247, 00166 Rome, Italy; carlo.tomino@sanraffaele.it

**Keywords:** vericiguat, heart failure, worsening, vericiguat metabolism, vericiguat mechanism of action, NO-sGC pathway

## Abstract

Heart failure (HF) is a syndrome characterized by signs and symptoms resulting from structural or functional cardiac abnormalities, confirmed by elevated natriuretic peptides or evidence of congestion. HF patients are classified according to left ventricular ejection fraction (LVEF). Worsening HF (WHF) is associated with increased short- and long-term mortality, re-hospitalization, and healthcare costs. The standard treatment of HF includes angiotensin-converting enzyme inhibitors, angiotensin receptor–neprilysin inhibitors, mineralocorticoid-receptor antagonists, beta-blockers, and sodium-glucose-co-transporter 2 inhibitors. To manage systolic HF by reducing mortality and hospitalizations in patients experiencing WHF, treatment with vericiguat, a direct stimulator of soluble guanylate cyclase (sGC), is indicated. This drug acts by stimulating sGC enzymes, part of the nitric oxide (NO)–sGC–cyclic guanosine monophosphate (cGMP) signaling pathway, regulating the cardiovascular system by catalyzing cGMP synthesis in response to NO. cGMP acts as a second messenger, triggering various cellular effects. Deficiencies in cGMP production, often due to low NO availability, are implicated in cardiovascular diseases. Vericiguat stimulates sGC directly, bypassing the need for a functional NO-sGC-cGMP axis, thus preventing myocardial and vascular dysfunction associated with decreased sGC activity in heart failure. Approved by the FDA in 2021, vericiguat administration should be considered, in addition to the four pillars of reduced EF (HFrEF) therapy, in symptomatic patients with LVEF < 45% following a worsening event. Cardiac rehabilitation represents an ideal setting where there is more time to implement therapy with vericiguat and incorporate a greater number of medications for the management of these patients. This review covers vericiguat’s metabolism, molecular mechanisms, and drug–drug interactions.

## 1. Introduction to Heart Failure (HF) and to Worsening HF (WHF)

The guidelines from the American Heart Association (AHA), American College of Cardiology (ACC), and Heart Failure Society of America (HFSA) define HF as “a clinical syndrome marked by symptoms and signs arising from structural heart disease, heightened filling pressures, or increased levels of natriuretic peptides”. This definition specifically excludes individuals with asymptomatic structural heart disease who are deemed to be at risk for HF. According to the universal definition, HF is characterized by a clinical syndrome featuring signs and/or symptoms stemming from structural and/or functional cardiac abnormalities, which are corroborated by elevated natriuretic peptides and/or objective evidence of pulmonary or systemic congestion [1,2]. Patients with HF are categorized based on left ventricular ejection fraction (LVEF) into those with reduced EF (HFrEF, EF ≤ 40%), mildly reduced EF (HFmrEF, EF 41–49%), preserved EF (HFpEF, EF ≥ 50%), and improved EF (HFimpEF, baseline EF ≤ 40%, a ≥10-point increase in EF, and a second EF > 40%) [3].

**Figure 1:**
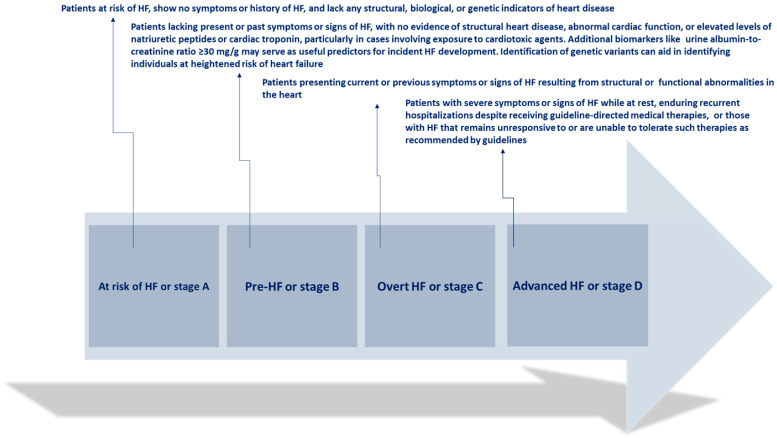
Clinical progression of heart failure. Adapted from Bozkurt, 2024 [4].

The clinical progression of HF is characterized by episodes of deteriorating symptoms and signs (Figure 1)

These instances of WHF are followed by an elevated risk of hospitalizations and mortality, imposing a significant strain on the healthcare system due to their frequency, urgency, and prognostic implications. WHF denotes the gradual decline in the heart’s capacity to efficiently pump blood, resulting in insufficient delivery of oxygen and nutrients to satisfy body demands. This deterioration typically presents with symptoms such as dyspnea, fatigue, fluid retention, and diminished exercise tolerance, and it can manifest gradually over time or abruptly during acute exacerbations [5].

The pathophysiology underlying WHF involves a decline in the heart pumping function. Common culprits include chronic conditions like coronary artery disease, hypertension, and cardiomyopathies. As the heart’s strength diminishes, it struggles to expel blood effectively, leading to fluid accumulation in the lungs and peripheral tissues. Neurohormonal systems, such as the renin–angiotensin–aldosterone system and the sympathetic nervous system, often become activated in an effort to compensate, but these mechanisms can contribute to further cardiac damage and worsening function. Additionally, inflammatory processes and structural alterations in the heart may also contribute to the progression of heart failure [5].

The management of WHF has traditionally relied on hospital-based care. However, the growing prevalence of HF and its associated costs on healthcare systems have prompted the development of alternative approaches to prolonged hospitalizations [6]. To mitigate the risk of HF hospitalizations and mortality, guidelines advocate for the use of angiotensin-converting enzyme inhibitors (ACEIs), angiotensin receptor–neprilysin inhibitors (ARNIs), mineralocorticoid receptor antagonists, beta-blockers, and sodium–glucose co-transporter 2 (SGLT-2) inhibitors. Recent analyses also demonstrate the effectiveness of guideline-recommended medical therapy (GRMT) in preventing outpatient WHF events, such as emergency visits requiring intravenous diuretic administration and outpatient visits requiring diuretic dose intensification [7].

**Figure 2:**
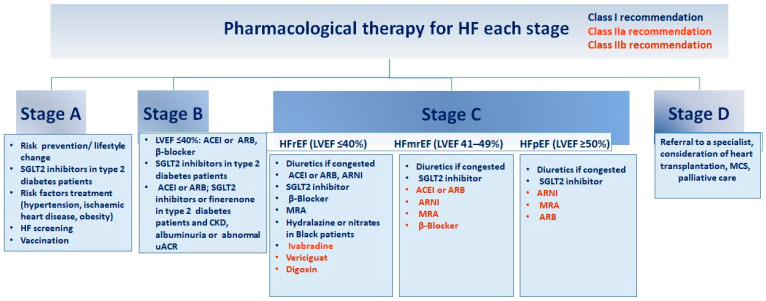
Guidelines for pharmacological intervention at every phase of heart failure. Recommendations for pharmacological therapy across the spectrum of HF stages, from risk prevention to advanced HF (stages A to D), are delineated below. Each treatment is color-coded based on the class of recommendation and level of evidence as outlined in HF guidelines. ACEI (angiotensin-converting enzyme inhibitor), ARB (angiotensin receptor blocker), ARNI (angiotensin receptor–neprilysin inhibitor), CKD (chronic kidney disease), GDMT (guideline-directed medical therapy), HFmrEF (HF with mildly reduced ejection fraction), HFpEF (HF with preserved ejection fraction), HFrEF (HF with reduced ejection fraction), LVEF (left ventricular ejection fraction), MCS (mechanical circulatory support), MRA (mineralocorticoid receptor antagonist), SGLT2 (sodium–glucose cotransporter 2), uACR (urine albumin-to-creatinine ratio). Adapted from Bozkurt, 2024 [4].

Different drugs are being studied for the management of WHF (Figure 2). A recent study extensively reviews various trials and addresses the management of different symptoms, such as congestion. It also discusses the use of remote monitoring technologies [8].

New data emerged from the VICTORIA (Vericiguat Global Study in Subjects with Heart Failure with Reduced Ejection Fraction) trial, which enrolled patients with LVEF <45%, New York Heart Association (NYHA) Classification class II–IV, elevated natriuretic peptide concentrations, and WHF. WHF was defined as an HF hospitalization within 6 months before randomization or an episode of decompensation with outpatient treatment using intravenous furosemide 3 months before randomization [8,9]. The study identified a high-risk group, where the annual event rate of cardiovascular death or HF hospitalizations was 37.8 events per 100 patient-years for those on placebo and 33.6 events per 100 patient-years for those on vericiguat. The 10% relative risk reduction of the primary endpoint (HR 0.90, 95% CI 0.82–0.98) corresponded to a 3.7 absolute risk reduction, which was similar in magnitude to that reported by previous trials [9,10]. The benefit of vericiguat did not significantly vary across different levels of risk in WHF and across the range of times from WHF to randomization. Based on these results, clinicians should consider vericiguat administration in addition to the four pillars of HFrEF therapy, in symptomatic patients with LVEF < 45% following a WHF event [11,12]. Interestingly, a recent review highlights that vericiguat has the potential to target an additional pathway in heart failure, particularly for older and frail patients showing clinical worsening. Due to its favorable safety profile, it may be easier to manage vericiguat than standard therapies for this population [13]. Thus, studies evaluating possible hemodynamic changes induced by vericiguat in HFrEF patients who demonstrated WHF despite treatment with the four foundational guideline-recommended therapies show that vericiguat decreases the pulmonary artery wedge pressure (PAWP) and preserves cardiac output in both the acute and later phases without changes in systemic vascular resistance (SVR), pulmonary vascular resistance (PVR), and cardiac index (CI) in either phase. These findings differ from the hemodynamic effects of Riociguat, a first lead soluble guanylate cyclase (sGC), which is a stimulator developed for two different forms of pulmonary hypertension (PH), in HFrEF patients, which showed a reduction in both SVR and PVR, and an increase in CI without changes in PAWP [14]. The consistent reduction in PAWP underscores the well-tolerated nature of vericiguat and its potential to enhance cardiac performance in HFrEF patients. The consistent reduction in PAWP highlights vericiguat’s well-tolerated nature and its potential to improve cardiac performance in HFrEF patients.

Furthermore, exercise rehabilitation appears to decrease the likelihood of subsequent HF events in older, frail patients admitted for decompensated HF, particularly in individuals who adhere closely to the exercise [12,15,16]. In addition, the new updated 2024 Cochrane review, which provided additional evidence from 16 randomized trials, supported the conclusions of the previous 2018 version, i.e., that exercise-based cardiac rehabilitation (ExCR) likely reduces the risk of all-cause hospital admissions and heart failure-related hospital admissions, and may result in important improvements in the health-related quality of life (HRQoL) [17].

## 2. Vericiguat

**Figure 3:**
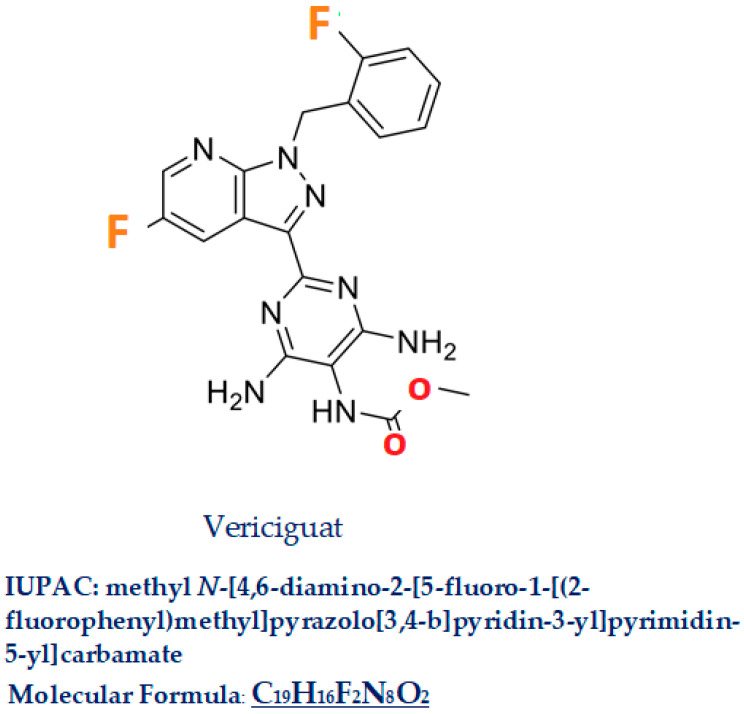
Vericiguat.

Vericiguat, commercial name Verquvo/BAY 1021189, a compound with a molecular weight of 426.4, is a 5-fluoro-1H-pyrazolo[3,4-b]pyridine (IUPAC name: methyl *N*-[4,6-diamino-2-[5-fluoro-1-[(2-fluorophenyl)methyl]pyrazolo[3,4-b]pyridin-3-yl]pyrimidin-5-yl] carbamate C_19_H_16_F_2_N_8_O_2_ Figure 3). In this molecule, the amino hydrogen at position 1 has been replaced by a 2-fluorobenzyl group, and the hydrogen at position 3 has been substituted by a 4,6-diamino-5-[(methoxycarbonyl)amino] pyrimidin-2-yl group [18]. Vericiguat was approved by the FDA (first approved on 19 January 2021) [19], then by EMA (16 July 2021) [20], and by the Pharmaceuticals and Medical Device Agency in Japan in 2021 [21] for HFrEF. Similar drugs are Dapagliflozin, Riociguat, Sacubitril/Valsartan.

Vericiguat is characterized as a weakly basic drug with low water solubility and high intestinal permeability, classified as class II according to the Biopharmaceutics Classification System [22].

**Figure 4:**
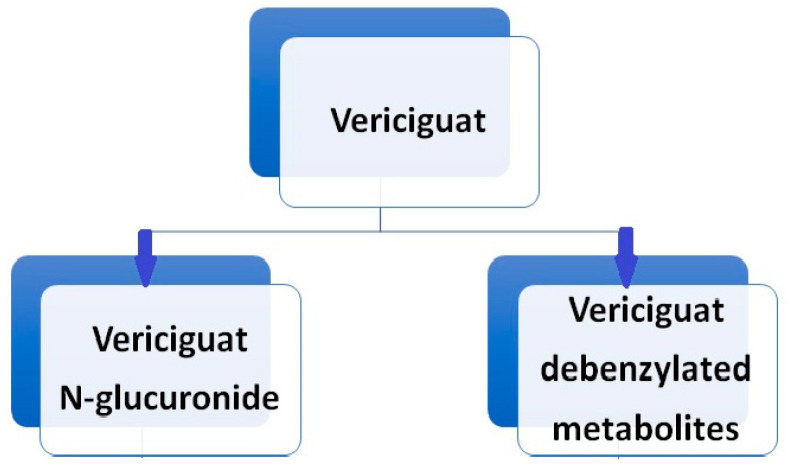
Vericiguat metabolism.

Vericiguat undergoes primarily glucuronidation, facilitated by uridine diphosphate-glucuronosyltransferase (UGT 1A9 and UGT 1A1), leading to the formation of N-glucuronide M-1, which lacks pharmacological activity against sGC. A minor fraction of the drug (<5%) undergoes metabolism via the P450 (CYP450) clearance pathway (Boettcher et al., 2020 [22]) (Figure 4). While sex and body weight are significant covariates in vericiguat’s volume of distribution, age, ethnicity, bilirubin, estimated glomerular filtration rate (eGFR), and albumin concentration do not affect the pharmacokinetics of vericiguat [23,24].

Following the oral administration of radiolabeled vericiguat, approximately 53% of the administered radioactivity was recovered in the urine and 45% in the feces. A human mass balance study revealed that the urine portion comprised approximately 40.8% N-glucuronide metabolite, 7.7% other metabolites, and 9% unchanged parent drug, while virtually the entire portion recovered in the feces consisted of unchanged vericiguat. Vericiguat is a low-clearance drug, with an observed plasma clearance of 1.6 L/h in healthy volunteers and 1.3 L/h in patients with systolic heart failure. The half-life of vericiguat is 30 h in patients with HF [25].

The adverse side effects of vericiguat primarily include symptomatic hypotension due to vasorelaxation, syncope, and anemia. Changes in vasoactive hormones for cyclic guanosine monophosphate (cGMP), plasma renin activity, and noradrenaline, as well as a decrease in creatinine, urea, and uric acid levels, have also been observed.

Vericiguat stimulates directly the sGC enzyme [26] that amplifies the effects of nitric oxide (NO) [27]. In HF, a reduced NO availability can be observed, due to a reduced endothelial nitric oxide synthase (eNOS) activity, secondary to inflammation and an increase in TNFα production [28]. Keeping sufficient levels of NO is essential to maintain normal cardiac contractility and vascular tone [29], as it acts by binding the heme-containing domain of the sGC enzyme and directly stimulates the production of cGMP which, in turn, exerts several effects on vascular cells, regulating endothelial permeability, cell growth, vasomotor tone, and interactions with circulating blood cells [30]. Along with a direct stimulation of sGC, vericiguat also increases the positive effects of NO, by stabilizing the nitrosyl–heme complex [31], thus resulting in the restoration of the NO-sGC-cGMP pathway even in a low-NO-level microenvironment.

Below, we will describe in detail the components of the NO-sGC-cGMP pathway and the synergistic effect of vericiguat.

### 2.1. Nitric Oxide (NO)

**Figure 5:**
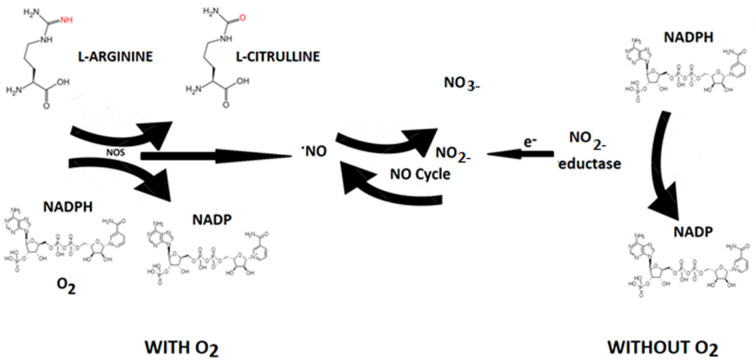
NO synthesis. In living organisms, NO can be produced by L-arginine through the action of NO synthase (NOS) through the consumption of reduced nicotinamide adenine dinucleotide phosphate (NADPH+) and oxygen that converts L-arginine to L-citrulline with NO production. NOS-independent NO formation can be interpreted as a contingency mechanism, ensuring adequate NO production in situations of limited oxygen supply.

Nitric oxide (NO) is an endogenous vasodilator produced by various cells in the body by a family of enzymes called nitric oxide synthases [NOS, NOS III, and NOS3) by the oxidation of L-arginine (L-Arg) to L-citrulline (Figure 5).

Nitric oxide synthase is present in three isoforms, known as neuronal (nNOS or NOS1), inducible (iNOS or NOS2), and endothelial (eNOS or NOS3). Both nNOS and eNOS are calcium-dependent enzymes that are constitutively expressed in various cells throughout the body, producing picomolar or nanomolar amounts of NO. iNOS, also found in multiple cell types, is induced in immunological or inflammatory conditions, typically triggered by IL1β, TNF, and LPS, resulting in the generation of micromolar or millimolar levels of NO. Nitrate can be converted to nitrite by nitrate reductase produced by oral cavity bacteria. Nitrite, in turn, is reduced to NO by nitrite reductases, which are oxygen-independent enzymes such as xanthine oxidoreductase, deoxyhemoglobin, and deoxymyoglobin. Nitrite reductase generates NO that induces vasodilation in the cardiovascular system, thereby promoting cell survival by increasing blood flow even under hypoxic conditions [32]. The half-life of NO is very brief (approximately 2 milliseconds in the blood and 2 s in the tissues). Nevertheless, its steady-state concentration is a crucial factor in determining its biological function. Different cellular targets respond to varying concentrations of NO. For instance, processes mediated by cGMP are activated when NO concentration is below 1–30 nM, while stabilization of hypoxia-inducible factor-1α (HIF-1α) happens at NO concentrations ranging between 100 and 300 nM. NO can be viewed as both an autocrine and paracrine signaling molecule, with its lifespan and diffusion gradients constrained by scavenging reactions involving hemoglobin, myoglobin, and other radicals. Nevertheless, NO can be stabilized in the bloodstream and tissues through oxidation to nitrate and nitrite. These compounds can be regarded as endocrine molecules that circulate in the blood, accumulate in tissues, and possess the potential to be converted back to NO under both physiological and pathological conditions [32].

The identification and characterization of the complex machinery responsible for NO generation by endothelial cells, along with its role in endothelium-dependent vascular relaxation in 1990 by Salvador Moncada [33], represent a significant milestone in defining the fundamental mechanisms regulating vascular physiology and regional blood flow adjustment. The evidence supporting the pivotal role of NO, released by endothelial cells in the intrinsic relaxation of vascular smooth muscle cells, coupled with potent anti-platelet action, has been instrumental in understanding the pathophysiology of relevant disease states such as atherothrombosis, vascular injury, and heart failure [34]. Furthermore, the recognition of NO/cGMP signaling’s critical role in cardiac contraction and relaxation has contributed to understanding the intricate processes underlying myocardial dysfunction, encompassing both reduced and preserved ejection fraction [35,36].

In blood vessels, NO is continuously generated from L-arginine by the enzyme eNOS, which is the most abundant isoform of NOS (Figure 4). The continuous activation of eNOS is due to endothelial cells responding to forces induced by the streaming blood, such as shear stress, which triggers the phosphorylation of eNOS by the Ser/Thr protein kinase PKB/Akt. Additionally, the activation of endothelial cells by hormones or biogenic amines acting on G-protein-coupled receptors (GPCRs) increases endothelial-free Ca^2+^ levels. This signal stimulates eNOS activity in a Ca^2+^-calmodulin-dependent manner and contributes to the physiological adaptation controlling regional blood flow in tissues.

Among other significant effects of NO, there is vasodilation, which occurs primarily through a reduction in cytosolic Ca^2+^ levels, following the activation of sGC in the smooth muscle. Active sGC converts guanosine-5′-triphosphate (GTP) into cGMP, which binds to and activates protein kinase G (PKG). PKG I phosphorylates several proteins involved in cellular Ca^2+^ homeostasis, including 1,4,5-inositoltrisphosphate (IP3) receptor-associated cGMP kinase substrate (IRAG), blocking IP3-evoked Ca2+ release from the sarcoplasmic reticulum by inhibiting the IP3-receptor I in its membrane. Furthermore, PKG I phosphorylates the large-conductance calcium-activated potassium channels (BKCa K^+^-channels), increasing their open probability, and promoting potassium outflow and hyperpolarization, thereby reducing the effect of depolarizing signals.

Finally, NO activates the sarcoplasmic reticulum ATPase (SERCA), inducing sequestration from the cytosol into the SR, in a cGMP-independent manner through direct NO (and superoxide)-induced reversible S-glutathiolation at Cys674 of SERCA.

### 2.2. NO-sGC Pathway

Briefly, in vivo, the effects of NO are mediated through both cGMP-dependent and cGMP-independent pathways. In the cGMP-dependent pathway, NO signals are translated into increased cGMP levels by activating soluble guanylate cyclase. Elevated cellular cGMP, in turn, stimulates various protein kinases within the cell. These kinases then inhibit platelet aggregation, induce smooth muscle relaxation, and modulate cell growth and differentiation [37].

In the cGMP-independent pathway, the NO signal induces modifications to protein structure through S-nitrosylation, S-glutathionylation, and tyrosine nitration. These alterations in cellular protein conformations result in changes in functional activity. The NO–sGC–cGMP pathway is a critical signaling mechanism involved in cardiovascular, cardio-pulmonary, and cardiorenal regulation. sGC comprises α- and β-subunits with a NO-binding structure. NO after diffusion in the cytosol across cell membranes binds to cytosolic sGC, inducing a conformational change in the enzyme and activating its catalytic site, which converts guanosine triphosphate to cGMP. cGMP then binds to and activates cGMP-activated protein kinases, cGMP-regulated ion channels, and cGMP-regulated phosphodiesterases (PDEs). This pathway plays a crucial role in regulating vascular tone and maintaining tissue homeostasis through various mechanisms, including anti-fibrotic and anti-inflammatory effects [37,38].

The dysregulation of the NO–sGC–cGMP signaling pathway can occur through oxidative stress, which alters the redox state of sGC, leading to the formation of oxidized and heme-free sGC (apo-sGC), rendering it unresponsive to NO. Endothelial dysfunction can also impair NO production, resulting in decreased NO bioavailability and reduced tissue cGMP levels. Additionally, other factors contributing to impaired NO–sGC–cGMP signaling include decreased levels of L-arginine due to arginase activity, the limited availability of L-arginine, the downregulation of nitric oxide synthase (NOS) in the vascular endothelium, NO inactivation by superoxide anion, elevated plasma concentrations of the endogenous NOS inhibitor ADMA (asymmetric dimethylarginine), disrupted sGC transcription, and the reduced stability of sGC mRNA. These dysfunctions can lead to various cardiovascular, cardiopulmonary, and cardiorenal diseases, such as pulmonary arterial hypertension (PAH) and other forms of pulmonary hypertension [37,38,39]. Acute inflammation can also have an effect on NO levels, as an increased concentration of TNFα can reduce eNOS activity [28].

### 2.3. Vericiguat Molecular Mechanism

**Figure 6:**
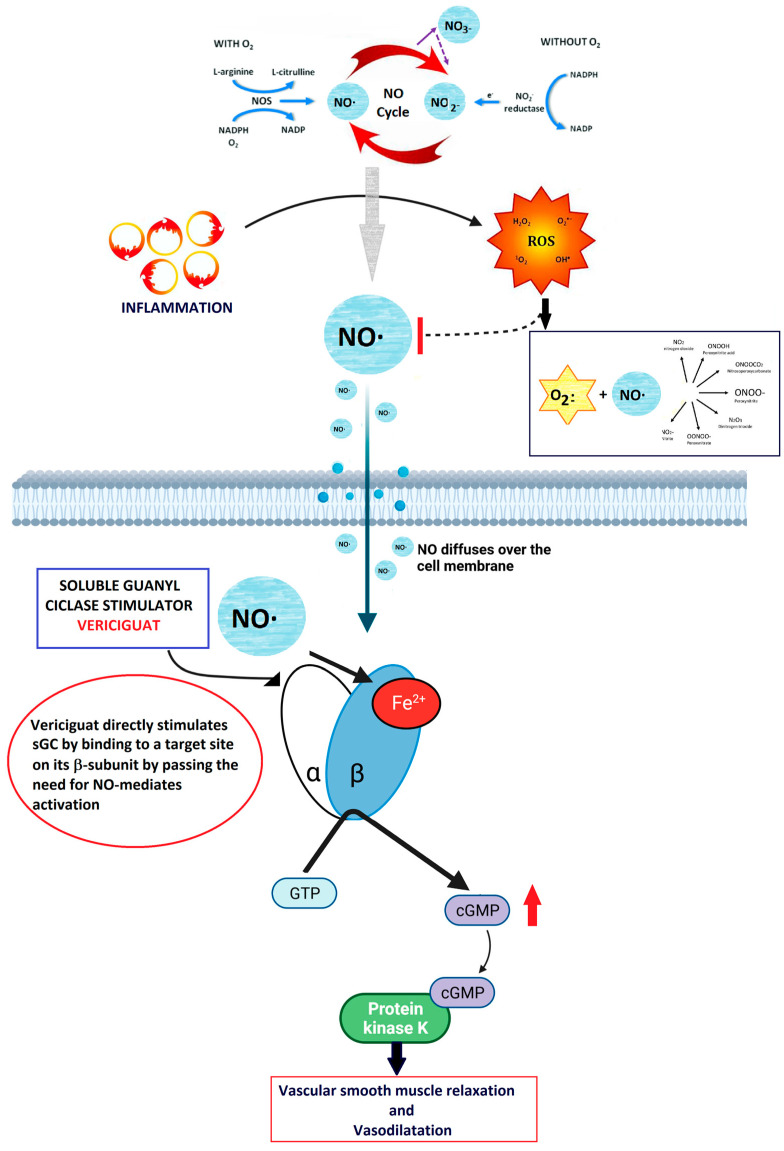
Summary of the mechanism of action of vericiguat. NO is produced in the endothelium and diffuses into the smooth muscle, where it binds to sGC. This binding causes a conformational change that catalyzes the production of cGMP. The cGMP then activates downstream kinases, leading to vasodilation. sGC stimulators and activators, including NO, bind to the reduced heme (Fe2+) form of sGC. This interaction catalyzes the conversion of GTP to cGMP, resulting in vasodilation. Vericiguat directly stimulates sGC by binding to a specific site on its β-subunit.

As reported above, under physiological conditions, the vascular endothelium produces NO, which in turn stimulates the sGC-mediated synthesis of cGMP [38]. The endocardial endothelium, responsive to NO, modulates contractility and diastolic function by elevating cGMP levels [40]. cGMP activation leads to the activation of PKG, initiating downstream pathways that promote vasodilation, reduce inflammation and fibrosis, inhibit hypertrophy, and diminish cardiac remodeling [41]. Vericiguat’s significance in HF treatment stems from the hindrance of NO-dependent intracellular cGMP generation due to ongoing inflammation, oxidative stress, and endothelial dysfunction, which lower NO levels [42] (Figure 6).

This enzyme is crucial for cGMP generation in cardiac and vascular tissues, serving as a messenger to facilitate cardiac and vasorelaxation via phosphokinase G. Studies have revealed cGMP deficiency in both HF with reduced ejection fraction (HFrEF) and HF with preserved ejection fraction (HFpEF) [43]. Vericiguat additionally stabilizes nitric oxide at its binding site, enhancing sGC sensitivity to nitric oxide [44].

cGMP is also modulated by additional signaling pathways. Natriuretic peptides (NPs), including atrial natriuretic peptides and B-type natriuretic peptides, increase cGMP levels by activating membrane-bound guanylate cyclase, particularly guanylate cyclase, pGC [44].

### 2.4. Drug–Drug Interactions

The administration of omeprazole, a proton pump inhibitor (PPI) used in acid reflux treatment, has been shown to decrease the absorption of vericiguat. However, drug–drug interaction studies suggest that vericiguat is suitable for managing patients with heart failure and multiple comorbidities requiring multiple medications. It is important to note that if a patient is taking a long-acting nitrate, guanylate cyclase stimulator, or phosphodiesterase-5 (PDE5) inhibitor, vericiguat should be avoided to prevent syncope and hypotension. Additionally, patients with severe anemia should avoid vericiguat due to concerns that the drug may decrease hemoglobin levels [22].

### 2.5. Implementation of Personalized Therapy

Guideline-directed medical therapy (GDMT) reduces cardiovascular mortality and morbidity and enhances the quality of life for patients with HF and HFrEF. However, despite the recent advancements in pharmacotherapy for WHF, as described above, effectively implementing GDMT in clinical practice remains challenging [45]. Besides clinical inertia, clinical conditions such as hypotension, impaired renal function, hyperkalemia, and polypharmacy—which contraindicate therapy and/or are associated with low tolerability—may partially explain the poor implementation of GDMT frequently observed in HFrEF patients. At the patient level, non-adherence to cardiovascular pharmacotherapy is the primary barrier. Approximately 30–75% of older adults do not take their medications as prescribed, 50% of prescriptions are known to be taken incorrectly, and 33–69% of drug-related hospital admissions are due to patients not following their medication regimens. Non-adherence is exacerbated by factors such as polypharmacy, multimorbidity, physical or cognitive impairment, poor patient education, and the cost and complexity of treatment. This non-adherence is associated with poor quality of life (QoL), increased hospitalizations, mortality, and higher medical costs. Therefore, assessing adherence should be a routine part of patient care [46].

Importantly, a recent real-world study found that despite participants being older than those in clinical trials, adherence to and persistence with vericiguat were satisfactory across all age groups. Starting vericiguat resulted in an intensification of concomitant GDMT, although a better understanding of barriers to the up-titration of vericiguat and the implementation of other GDMT, particularly for women and elderly patients, requires further evidence [47]. Monitoring patient adherence requires significant time and resource allocation. Additionally, vericiguat necessitates a titration regimen, which includes two up-titration visits, spaced two weeks apart, to achieve the maximum dose. This process results in a total titration period of 28 days to increase the dosage from 2.5 mg to 10 mg. Cardiac rehabilitation (CR)—a comprehensive disease management program, strongly recommended by HF guidelines [48]—represents an ideal setting where there is more time to implement therapy and incorporate a greater number of essential medications for the management of these patients.

### 2.6. Cost–Utility of the Addition of Vericiguat

Few studies [49,50] evaluate the cost–utility of adding vericiguat for treating HF. All the studies conducted in China or for the VICTORIA trials highlight that despite the high cost of vericiguat, its treatment for CHF is not cost-effective. While clinical trials like VICTORIA have demonstrated its efficacy in reducing cardiovascular death and heart failure hospitalizations, the cost-effectiveness of this medication remains a crucial consideration. Initial analyses suggest that vericiguat may be cost-effective in certain patient populations, particularly those at a higher risk of heart failure events. The drug’s ability to reduce hospitalizations could potentially offset its acquisition costs by decreasing healthcare utilization. However, the cost-effectiveness varies depending on factors such as the specific patient population, local healthcare costs, and willingness-to-pay thresholds. Some studies have indicated that vericiguat may be more cost-effective in patients with more severe heart failure or those who have had recent hospitalizations. The long-term economic impact of vericiguat use is still being evaluated, as more real-world data become available. Healthcare systems and payers must carefully consider these economic analyses alongside clinical efficacy data when making decisions about formulary inclusion and reimbursement. As with many novel therapies, the cost-effectiveness of vericiguat may improve over time if its price decreases or if additional benefits are discovered through ongoing research and clinical experience.

## 3. Conclusions

Vericiguat is a critical treatment option for high-risk patients with HFrEF who are already receiving guideline-directed medical therapy and have recently experienced worsening heart failure. Targeting the cGMP pathway presents a distinctive mechanism of action, potentially enhancing clinical outcomes for patients with HFrEF.

As awareness of the clinical and prognostic significance of WHF grows, vericiguat effectively fills an important therapeutic gap by reducing the incidence of cardiovascular death and WHF in high-risk HFrEF patients. The possibility of expanding the use of vericiguat to a broader range of patients is presently investigated by the VICTOR study, a phase III randomized, placebo-controlled clinical trial, on the effectiveness and safety of the sGC stimulator vericiguat/MK-1242 in adults diagnosed with HFrEF who have not been hospitalized for heart failure recently or required outpatient intravenous diuretics [48]. Table 1 reports and compares the two trials: VICTORIA versus VICTOR. The latter is an active study, not recruiting with an estimated study completion date of 15 June 2025, and as of 10 July 2024, no results are reported on ClinicalTrials.gov [ID: NCT05093933].

Ongoing research and clinical insights will continue to clarify the role of vericiguat in managing HF and its potential to alleviate the healthcare burden associated with this condition. Through continued study and development, vericiguat holds promise to transform the treatment approach for HF, offering new possibilities for enhanced outcomes and quality of life for patients globally.

## Figures and Tables

**Table 1:** Worsening HF included in clinical trial study designs: VICTORIA trial versus VICTOR trial.

	VICTORIA [9] (Patients: 5050)	VICTOR [48] (Patients: 6000)
	Vericiguat 2526 female 1921 male Placebo 2524 female 1921 male	Ongoing
Study drug	Vericiguat vs. placebo	Vericiguat vs. placebo
Key inclusion criteria	Chronic HF with NYHA functional class II–IV EF < 45% Receiving HF GDMT Prior HF hospitalization within 6 months or outpatient intravenous diuretic for HF within 3 months Elevated NT-proBNP levels	Chronic HF with NYHA functional class II–IV EF ≤ 40% Elevated NT-proBNP levels
Primary endpoint	Time to First Occurrence of Composite Endpoint of Cardiovascular (CV) Death or Heart Failure (HF) Hospitalization	Time to First Occurrence of Composite Endpoint of Cardiovascular (CV) Death or Heart Failure (HF) Hospitalization The first event of CV death or HF hospitalization as confirmed by a clinical event committee (CEC)
Select secondary endpoints	Time to CV death Time to first HFH Time to total (first and recurrent) HFHs Time to total first occurrence of composite of all-cause mortality or HFH Time to all-cause mortality Safety and tolerability	Time to first occurrence of CV First event of CV death as confirmed by CEC Time to first occurrence of HF hospitalization The first event of HF hospitalization as confirmed by CEC Time to total HF hospitalizations, all events of HF hospitalization as confirmed by CEC Safety and tolerability
Randomized Double-blind 1:1	2.5 mg od/5 mg od/10 mg od (up-titration at 2-week intervals) Placebo	2.5, 5.0, or 10.0 mg orally once daily Placebo
Median follow-up	10.8 months	40.0 months

## Data Availability

Not applicable.
